# Generalized gametic relationships for flexible analyses of parent-of-origin effects

**DOI:** 10.1093/g3journal/jkab064

**Published:** 2021-03-10

**Authors:** Norbert Reinsch, Manfred Mayer, Inga Blunk

**Affiliations:** Institute of Genetics and Biometry, Leibniz-Institute for Farm Animal Biology, 18196 Dummerstorf, Germany

**Keywords:** imprinting variance, genomic imprinting, maternal effects, gametic relationships

## Abstract

A class of epigenetic inheritance patterns known as genomic imprinting allows alleles to influence the phenotype in a parent-of-origin-specific manner. Various pedigree-based parent-of-origin analyses of quantitative traits have attempted to determine the share of genetic variance that is attributable to imprinted loci. In general, these methods require four random gametic effects per pedigree member to account for all possible types of imprinting in a mixed model. As a result, the system of equations may become excessively large to solve using all available data. If only the offspring have records, which is frequently the case for complex pedigrees, only two averaged gametic effects (transmitting abilities) per parent are required (reduced model). However, the parents may have records in some cases. Therefore, in this study, we explain how employing single gametic effects solely for informative individuals (*i.e.*, phenotyped individuals), and only average gametic effects otherwise, significantly reduces the complexity compared with classical gametic models. A generalized gametic relationship matrix is the covariance of this mixture of effects. The matrix can also make the reduced model much more flexible by including observations from parents. Worked examples are present to illustrate the theory and a realistic body mass data set in mice is used to demonstrate its utility. We show how to set up the inverse of the generalized gametic relationship matrix directly from a pedigree. An open-source program is used to implement the rules. The application of the same principles to phased marker data leads to a genomic version of the generalized gametic relationships.

## Introduction

Epigenetic marks of parent-of-origin may lead to the functional inequivalence of parental alleles with respect to gene expression. This pattern is known as genomic imprinting. According to previous reviews (*e.g.*, Lawson *et al.* 2013), the phenotypic signature of genomic imprinting at a single locus appears as a trait mean difference between reciprocal heterozygotes AB and BA (the first allele is paternal). The broader term parent-of-origin effects (POEs) is often used to emphasize that other underlying mechanisms such as maternal genetic effects could contribute to the observed inequivalence (Hager et al. 2008). For complex traits, there is evidence that variants of nonimprinted genes can generate substantial POEs by interacting with imprinted loci (Mott *et al.* 2014), despite the small number known for the latter. An imprinting variance component can serve as a type of joint phenotypic signature of multiple imprinted loci that could potentially affect a quantitative trait. This component summarizes the squared trait differences between reciprocal heterozygotes at all imprinted loci as a single quantity and it can be estimated by analyzing large pedigreed populations with suitable mixed models. Recent examples of this type of study in livestock genetics by Neugebauer *et al.* (2010a, 2010b), Tier and Meyer (2012), Blunk *et al.* (2017a, 2017b), Okamoto *et al.* (2019), and Inoue *et al.* (2020) all considered carcass traits in cattle. Furthermore, Blunk *et al.* (2020) investigated type 1 diabetes and rheumatoid arthritis in the field of human genetic epidemiology.

Some of the studies mentioned above used simplified relationships, including only sire and maternal grandsire information, mainly due to partially missing pedigree information (Okamoto *et al.* 2019) or to ensure that the number of equations was small when analyzing large volumes of data (Blunk *et al.* 2017a). In other studies, the mixed model employed a classical gametic relationship matrix (Tier and Meyer 2012; Blunk *et al.* 2020) derived from complete pedigree information for several tens to hundreds of thousands of individuals. The gametic relationship model is the most costly variant in terms of the number of equations for a given pedigree, but it allows imprinting analysis to be conducted in every case where mixed model analysis of pedigreed data is possible.

Shortly after its discovery, researchers recognized that the gametic relationship matrix (Smith and Allaire 1985; Schaeffer *et al.* 1989) is useful for isolating fractions of the genetic variance in quantitative traits caused by genomically imprinted loci. In the early stages of pedigree-based imprinting analysis, animal models were augmented by an additional vector of paternal (alternatively, maternal) gametic effects, which was usually modeled as uncorrelated with any other effect. In standard practice, the vector’s variance was represented as the product of a gametic relationship matrix and a variance component caused by polymorphisms at loci with only paternal (maternal) gene activity. These models were pioneered by De Vries et al. (1994) and used for more than a decade. However, they can account only for a single type of classical imprinting, where either the maternal or paternal alleles are fully silenced. A proposal by Hill and Keightly (1988) to consider both types of imprinting simultaneously was not implemented in any pedigree-based analyses of empirical data. Furthermore, there was uncertainty regarding the methods used to account for the effects of partially imprinted loci, where both alleles are active, but at different strengths depending on their parental origins.

A model for parent-of-origin analysis was subsequently developed (Neugebauer *et al.* 2010a, 2010b) and it is comprehensive in the sense that it accounts for all types of imprinting, *i.e.*, full or partial, and maternal or paternal (Blunk and Reinsch 2014). This so-called *reduced imprinting model* relates observations from nonparents (final progeny, *e.g.*, animals used for meat) to the transmitting abilities (half of the breeding values) of their parents. There are two correlated genetic effects per parent comprising the transmitting ability as sire and transmitting ability as dam, which reflect an animal’s genetic effect on its offspring under a paternal or maternal imprinting pattern. These two genetic effects are different in the presence of genomically imprinted loci. The variance in these differences is referred to as the imprinting variance because it summarizes the contributions from all types of possible imprinted loci. A numerator relationship matrix is necessary for parents only because the final progeny with observations, but without offspring, do not appear in the underlying pedigree and the resulting relationship matrix. The null hypothesis comprising the absence of polymorphic imprinted loci with an effect on the trait under investigation (*i.e.*, a zero imprinting variance) can be tested with a restricted maximum likelihood (REML) ratio test (RLRT).

Alternatively, a comprehensive *gametic model* can be used to estimate the same set of genetic covariances, including the imprinting variance (Tier and Meyer 2012; Meyer and Tier 2012), where one must estimate four gametic effects per individual, *i.e.*, two as sire and two as dam, and the relationships include the final progeny with observations. The *gametic model* allows for records from parents, which is an advantage compared with the *reduced imprinting model*. Furthermore, it is possible to extend this model with maternal genetic effects.

According to the principle applied, this maternal genetic component of the variance is a particular challenge because it is difficult to separate from the imprinting variance. Okamoto *et al.* (2019) showed that the resulting imprinting variance estimated with a model variant that uses information only for the sire and maternal grandsire (Blunk *et al.* 2017a; Okamoto *et al.* 2019) can also be interpreted as maternal genetic variance. Similarly, for the *reduced imprinting model*, it is possible to show that the imprinting variance and maternal genetic variance cannot be disentangled when both are present, and thus it is only possible to infer a composite component of variance (for a theoretical derivation, see Appendix A4).

The utilization of measured genotypes in genomic best linear unbiased prediction (gBLUP) models that include imprinting effects was outlined by Nishio and Satoh (2015). The first of the two variants of the proposed model (GBLUP-I1) contains an imprinting effect at all markers, which is modeled as independent of the actions of all markers as un-imprinted Mendelian loci. An additive genetic effect summarizes the latter per marker. The second model (GBLUP-I2) considers a paternal and maternal gametic effect with zero mutual correlation. Although not considered by Nishio and Satoh (2015), it is clearly possible to change this into a comprehensive model by abandoning the assumption of a zero correlation and by replacing pedigree-derived gametic relationships with a genomic counterpart of equal size and structure. In cases where not all pedigreed individuals are genotyped, this would then allow combined analysis of the genotyped and un-genotyped individuals in a single-step approach (Legarra *et al.* 2009; Aguilar *et al.* 2010; Christensen and Lund 2010). By contrast, it is not possible to easily extend the first model (GBLUP-I1) to a pedigree-derived counterpart.

The disadvantage of the *gametic model* is the large number of equations (Smith and Allaire 1985) used to represent the random genetic effects, particularly when estimating the variance components. A pedigree with a size of approximately half a million is a technical barrier for REML estimation in animal models using the currently available software packages (Shor *et al.* 2019). With a gametic parent-of-origin model, only a quarter of individuals require the same number of equations. Therefore, the question arises whether an option is available where models retain the flexibility of the *gametic model* while allowing for a considerably smaller number of equations for random genetic effects, and it must be as close as possible to the *reduced imprinting model*.

As a solution, we propose a generalization of the *gametic model* with a much smaller redefined vector of genetic effects obtained by linear transformation of the original gametic effects. We refer to the corresponding relationship matrix as a generalized gametic relationship matrix and present rules for its rapid inversion from the pedigree. As a result, the size of the *gametic model* becomes more manageable while retaining all of its advantages. We also show how the same type of transformation can be applied to measured genotypes to obtain conformable genomic and pedigree-derived versions of the new relationship matrix. In the following, we theoretically derive the generalization of the *gametic model*. Worked examples (available in the Supplementary material) are provided as illustrations for all of the models described. Finally, pedigreed mouse data are employed to demonstrate the utility of the *generalized gametic model* and to allow for conclusions regarding the influence of POEs on body mass.

## Materials and methods

### Theory

#### Generalized gametic relationships:

In gametic models under Mendelian inheritance, each individual i is represented by the additive genetic effects of its paternal gamete $g_{i,1}$ and maternal gamete $g_{i,2}$ (Schaeffer *et al.* 1989). These effects are usually arranged in a pairwise manner in a vector g of length 2t, which is twice the number t of individuals in the pedigree. The equation for a phenotypic observation $y_{i}$ of individual i is:

$$y_{i}=\mu_{i}+g_{i,1}+g_{i,2}+e_{i},$$

where $\mu_{i}={x^{\prime }}_{i}\beta$ is a place-holder for any combination of explanatory variables in vector ${x^{\prime }}_{i}$ with fixed effects β, and the residual $e_{i}$. Thus, the gametic model splits the additive genetic value (breeding value) $b_{i}$ of individual i into paternally derived and maternally derived parts: $b_{i}=g_{i,1}+g_{i,2}$.

The basic idea of reducing the equations in gametic models by a considerable number involves replacing the two gametic effects of a subset of u individuals by their pairwise average:

$$\frac{1}{2}\left(g_{i,1}+g_{i,2}\right)=a_{i},$$

which is known as the transmitting ability (half the breeding value) of individual i.

All gametic effects in the vector g can be arranged such that the gametic effects of all u individuals precede the gametic effects of those v that are bound in order to retain their distinct gametic effects for imprinting analysis, as explained in the next paragraph. The corresponding subdivision of g is:

$$g=\left[\begin{array}{c} g_{u}\\ g_{v} \end{array}\right].$$

The subvectors $g_{u}$ and $g_{v}$ have lengths of 2u and 2v, respectively. The covariances of all gametic effects in g are the elements of the 2t×2t gametic relationship matrix G (Schaeffer *et al.* 1989), which can be partitioned into sections that correspond to the relationships between the gametic effects in $g_{u}$ and $g_{v}$.

$$\mathrm{Var}\left[\begin{array}{c} g_{u}\\ g_{v} \end{array}\right]=\left[\begin{array}{cc} G_{uu} & G_{uv}\\ {G^{\prime }}_{uv} & G_{vv} \end{array}\right]=G$$

The required average gametic effects can be obtained by a linear transformation, which is defined by a matrix $K^{\prime }$ such that:

$$K^{\prime }g=\left[\begin{array}{cc} {K^{\prime }}_{u} & 0_{1}\\ 0_{2} & I_{v} \end{array}\right]\left[\begin{array}{c} g_{u}\\ g_{v} \end{array}\right]=\left[\begin{array}{c} a_{u}\\ g_{v} \end{array}\right]=a.$$

This operation replaces the gametic effects of all individuals in $g_{u}$ by their transmitting abilities in $a_{u}$. The upper-left partition ${K^{\prime }}_{u}$ of the transformation matrix $K^{\prime }$ has dimensions of u×2u, and it is defined as the Kronecker product of a u×u identity matrix $I_{u}$ and a row vector with two elements equal to $\frac{1}{2}$:

$${K^{\prime }}_{u}=I_{u}⊗\left[\begin{array}{cc} \frac{1}{2} & \frac{1}{2} \end{array}\right].$$

Furthermore, $K^{\prime }$ comprises a 2v×2v identity matrix $I_{v}$ and two null matrices, $0_{1}$ and $0_{2}$, with dimensions of u×2v and 2v×2u, respectively.

The covariance matrix of the transformed vector of gametic effects a then becomes:

$$\mathrm{Var}\left[\begin{array}{c} a_{u}\\ g_{v} \end{array}\right]=K^{\prime }GK=\bar{G},$$

which is called a generalized gametic relationship matrix in the following. In the context of imprinting analyses, a natural choice involves retaining the gametic effects of all individuals with their own phenotypes in vector $g_{v}$ and representing all their ancestors without records by their transmitting abilities as $a_{u}$. The subdivisions of $\bar{G}$ are then:

$$\bar{G}=\left[\begin{array}{cc} {K^{\prime }}_{u}G_{uu}K_{u} & {K^{\prime }}_{u}G_{uv}I_{v}\\ I_{v}{G^{\prime }}_{uv}K_{u} & I_{v}G_{vv}I_{v} \end{array}\right]=\left[\begin{array}{cc} \frac{1}{2}A_{u} & S_{uv}\\ {S^{\prime }}_{uv} & G_{vv} \end{array}\right].$$

The upper-left part $\frac{1}{2}A_{u}$ is equal to the coancestry matrix (half the numerator relationship matrix) of all ancestors without their own records, while $G_{vv}$ reflects the relationships between the gametic effects of all individuals with their own observations. Finally, $S_{uv}$ contains the covariances between the transmitting abilities and gametic effects. We consider a small example involving four individuals (IDs). There are three transmitting abilities for individuals 1, 2, and 3, with corresponding pairwise elements of $\frac{1}{2}$ in the transformation matrix $K^{\prime }$ and two gametic effects, where the elements in $K^{\prime }$ are one. The resulting generalized gametic relationship matrix $\bar{G}$ has dimensions of **5 **×** 5:**

$$\begin{array}{c} \begin{array}{cc} \begin{array}{ccc} \text{ID} & \text{sire} & \text{dam}\\ 1 & 0 & 0\\ 2 & 0 & 0\\ 3 & 1 & 2\\ 4 & 1 & 3 \end{array} & \;\;K^{\prime }=\left[\begin{array}{cccccccc} \frac{1}{2} & \frac{1}{2} & 0 & 0 & 0 & 0 & 0 & 0\\ 0 & 0 & \frac{1}{2} & \frac{1}{2} & 0 & 0 & 0 & 0\\ 0 & 0 & 0 & 0 & \frac{1}{2} & \frac{1}{2} & 0 & 0\\ 0 & 0 & 0 & 0 & 0 & 0 & 1 & 0\\ 0 & 0 & 0 & 0 & 0 & 0 & 0 & 1 \end{array}\right]\\ \bar{G}=\left[\begin{array}{ccccc} \frac{1}{2} & 0 & \frac{1}{4} & \frac{1}{2} & \frac{1}{4}\\ 0 & \frac{1}{2} & \frac{1}{4} & 0 & \frac{1}{4}\\ \frac{1}{4} & \frac{1}{4} & \frac{1}{2} & \frac{1}{4} & \frac{1}{2}\\ \frac{1}{2} & 0 & \frac{1}{4} & 1 & \frac{1}{4}\\ \frac{1}{4} & \frac{1}{4} & \frac{1}{2} & \frac{1}{4} & 1 \end{array}\right] \end{array} \end{array}.$$

#### Generalized gametic relationships in a gametic model:

Based on the descriptions above, the equation for an observation $y_{i}$ in a model that uses generalized gametic relationships and considers POEs remains the same as that in the classical *gametic model*:

$$y_{i}=\mu_{i}+g_{i,1}^{s}+g_{i,2}^{d}+e_{i},$$

where the superscripts s and d indicate the paternal ($g_{i,1}^{s}$) and maternal ($g_{i,1}^{d}$) expression patterns of the candidate’s gametes of paternal and maternal descent (indicated by subscripts 1 and 2), respectively. Gametes of the same parental descent, but opposite mode of expression ($g_{i,1}^{d}$ and $g_{i,2}^{s}$), are not directly related to any observation, but they can be estimated through their covariances with other effects. It is possible to average each pair of gametic effects with the same expression pattern from any individual without records, thereby obtaining the transmission abilities as sire $a_{i}^{s}=\frac{1}{2}\left(g_{{}_{i,1}}^{s}+g_{{}_{i,2}}^{s}\right)$ and dam $a_{i}^{d}=\frac{1}{2}\left(g_{{}_{i,1}}^{d}+g_{{}_{i,2}}^{d}\right)$.

A mixed model that considers POEs and uses the generalized relationship matrix then becomes:

$$Y=X\beta +Z_{s}a_{s}+Z_{d}a_{d}+e,$$

where Y is a vector of observations, β comprises the fixed effects, and X is the corresponding incidence matrix. The assumed covariance of random effects is:

$$\mathrm{Var}\left[\begin{array}{c} a_{s}\\ a_{d}\\ e \end{array}\right]=\left[\begin{array}{ccc} \bar{G}\sigma_{s}^{2} & \bar{G}\sigma_{sd} & 0\\ \bar{G}\sigma_{sd} & \bar{G}\sigma_{d}^{2} & 0\\ 0 & 0 & I\sigma_{e}^{2} \end{array}\right].$$

This *generalized gametic model* contains the size-reduced transformed gametic effect vectors $a_{s}$ and $a_{d}$, which are the counterparts of the full-size gametic effect vectors $g_{s}$ and $g_{d}$, respectively, and each has a length of 2t. Consequently, the model uses the corresponding relationship matrix $\bar{G}$ instead of the classical gametic relationships of G. The vector of genetic effects under a paternal (maternal) mode of expression $a_{s}$ ($a_{d}$) for genetic effects has an associated gametic variance component $\sigma_{s}^{2}$ ($\sigma_{d}^{2}$) and the covariance between the expression patterns is $\sigma_{sd}$. Thus, the imprinting variance (Neugebauer *et al.* 2010a, 2010b) is: $\sigma_{i}^{2}=\sigma_{s}^{2}+\sigma_{d}^{2}-2\sigma_{sd}$, which is equivalent to the variance in the differences between gametic effects under alternative modes of expression. Furthermore, the incidence matrices $Z_{s}$ and $Z_{d}$ link observations to the random gametic effects in $a_{s}$ and $a_{d}$, respectively, whereas no observation is linked to any of the transmitting abilities in the latter vectors. As a result, any incidence matrix $Z^{a}=\left[\begin{array}{cc} 0^{u} & Z^{v} \end{array}\right]$ that links observations to gametic effects in the generalized vector of genetic effects $a^{\prime }=\left[\begin{array}{cc} {a^{\prime }}_{u} & {g^{\prime }}_{v} \end{array}\right]$ can be considered a converted incidence matrix $Z^{g}=\left[\begin{array}{cc} 0^{2u} & Z^{v} \end{array}\right]$ from a classical gametic model that links the observations to the gametic effects in $g^{\prime }=\left[\begin{array}{cc} {g^{\prime }}_{u} & {g^{\prime }}_{v} \end{array}\right]$:

$$Z^{a}=Z^{g}K^{\prime }.$$

This transformation retains all columns in the partition $Z^{v}$, *i.e.*, one per gametic effect of individuals with phenotypes, and the number of null columns in $0^{u}$ of $Z^{a}$ collapses to half of that of $0^{2u}$ in $Z^{g}$. In the same manner, both incidence matrices $Z_{s}$ and $Z_{d}$ from the previous model equation are converted versions of their counterparts in the classical *gametic model*, which forms the basis for the proof of equivalence for the classical and generalized gametic models involving $\bar{G}$ (see Appendix A1). For a worked example based on a small data set analyzed with the *generalized gametic model*, see part 1 of the Supplementary material.

#### Reduced gametic model:

The *reduced imprinting model* initially described by Neugebauer *et al.* (2010a, 2010b) relates each observation from a final progeny i to the transmitting abilities as sire $a_{si}^{s}$ and dam $a_{di}^{d}$ for the parents si (sire of *i*) and di (dam of *i*), respectively. For a single observation $y_{i}$, we have the equation:

(1) $$y_{i}=\mu_{i}+a_{si}^{s}+a_{di}^{d}+r_{i},$$

where the residual $r_{i}$ is a sum of the Mendelian sampling effects of both parents ($m_{si}$ and $m_{di}$) and the measurement noise ($e_{i}$). The latter is identical to the residual of the gametic model. Thus, we have:

$$r_{i}=m_{si}+m_{di}+e_{i}.$$

Its variance is a function of the inbreeding coefficients $F_{si}$ and $F_{di}$ for the parents of i:

$$\mathrm{var}(r_{i})=\frac{1}{2}\left(1-F_{si}\right)\sigma_{s}^{2}+\frac{1}{2}\left(1-F_{di}\right)\sigma_{d}^{2}+\sigma_{e}^{2}.$$

After rewriting the transmitting abilities of the parents as the averages of the respective gametic effects, *i.e.*, $a_{si}^{s}=\frac{1}{2}\left(g_{1,si}^{s}+g_{2,si}^{s}\right)$ and $a_{di}^{d}=\frac{1}{2}\left(g_{1,di}^{d}+g_{2,di}^{d}\right)$, we obtain an equation in terms of gametic effects:

(2) $$y_{i}=\mu_{i}+\frac{1}{2}\left(g_{1,si}^{s}+g_{2,si}^{s}\right)+\frac{1}{2}\left(g_{1,di}^{d}+g_{2,di}^{d}\right)+r_{i}.$$

Then, the covariance of the gametic effects is:

$$\mathrm{Var}\left[\begin{array}{c} g_{s}\\ g_{d} \end{array}\right]=\left[\begin{array}{cc} \sigma_{s}^{2} & \sigma_{sd}\\ \sigma_{sd} & \sigma_{d}^{2} \end{array}\right]⊗G=\left[\begin{array}{cc} G\sigma_{s}^{2} & G\sigma_{sd}\\ G\sigma_{sd} & G\sigma_{d}^{2} \end{array}\right],$$

where the relationship matrix G of the gametic effects that define the transmitting abilities involved includes only the parents and their ancestors. The advantage of this *reduced gametic model* compared with the previously published version that uses only transmitting abilities and their relationship matrix $\frac{1}{2}A$ is that it allows us to easily integrate observations from parents by linking them to the respective gametic effects. Hence, for observations of any parent i, the observation equation becomes:

(3) $$y_{i}=\mu_{i}+g_{1,i}^{s}+g_{2,i}^{d}+e_{i}.$$

Part 1 of Supplementary A presents a worked example based on a small data set analyzed with the *reduced gametic model*.

#### Generalized reduced gametic model:

The disadvantage of the *reduced gametic model* is that it employs twice the number of equations compared with a version obtained using $\frac{1}{2}A$. However, for all individuals without their own records, it is possible to reduce the number of equations for random genetic effects by representing the individuals through their transmitting abilities (average gametic effects), while retaining separate gametic effects for all parents with phenotypes, *i.e.*, the vectors of gametic effects $g_{s}$and $g_{d}$ are replaced by appropriately transformed counterparts $a_{s}$ and $a_{d}$, respectively. Consequently, the covariance matrix of random genetic effects in a parsimonious *generalized reduced gametic model* that allows for parents with phenotypes is:

$$\mathrm{Var}\left[\begin{array}{c} a_{s}\\ a_{d} \end{array}\right]=\left[\begin{array}{cc} \bar{G}\sigma_{s}^{2} & \bar{G}\sigma_{sd}\\ \bar{G}\sigma_{sd} & \bar{G}\sigma_{d}^{2} \end{array}\right].$$

Furthermore, we need a diagonal matrix W of weights equal to $w_{i}=1$ for observations from parents to which observation equation (3) applies, and:

$$w_{i}={\left[\frac{\frac{1}{2}\left(1-F_{si}\right)\sigma_{s}^{2}+\frac{1}{2}\left(1-F_{di}\right)\sigma_{d}^{2}+\sigma_{e}^{2}}{\sigma_{e}^{2}}\right]}^{-1}$$

for the final progeny, where parents without their own records are represented by the transmitting abilities or both parents have a record and are represented by gametic effects [the equations are (1) and (2), respectively]. The same weight applies to mixed types of representations that arise in cases where one parent of the final progeny has a record whereas the other does not. The corresponding equations for observations $y_{i}$ of the final progeny are:

(4) $$y_{i}=\mu_{i}+a_{si}^{s}+\frac{1}{2}\left(g_{1,di}^{d}+g_{2,di}^{d}\right)+r_{i}$$

and

(5) $$y_{i}=\mu_{i}+\frac{1}{2}\left(g_{1,si}^{s}+g_{2,si}^{s}\right)+a_{di}^{d}+r_{i}.$$

For the *generalized reduced gametic model*, a detailed worked example based on a small data set is also presented in the Supplementary Part S1.

#### General model for parent-of-origin analyses:

A general comprehensive model for parent-of-origin analyses is based on the generalized gametic relationship matrix. Special cases of the generalized gametic relationship matrix $\bar{G}$ are the classical gametic relationship matrix $\bar{G}=G$ in the *gametic model* and $\bar{G}=\frac{1}{2}A$ in the *reduced imprinting model*. Correspondingly, the matrix W of weights can be an identity matrix that fits the classical *gametic model*, or a matrix of weights that differ from one like those in the *reduced model* for records of the final progeny. A general model can be specified for parent-of-origin analyses containing these two basic types of comprehensive imprinting models as well as models with any combination of gametic effects and transmitting abilities that can be obtained using our transformation matrix $K^{\prime }$. In matrix notation, the general model is:

$$Y=X\beta +Z_{s}a_{s}+Z_{d}a_{d}+\varepsilon ,$$

where ε is a vector of residuals, *i.e.*, $\varepsilon_{i}=e_{i}$ for records from individuals represented by two gametic effects or $\varepsilon_{i}=r_{i}$ for observations from final progeny linked to the genetic effects of their parents. The respective weights are:

$$w_{i}=1$$

and

$$w_{i}={\left[\frac{\frac{1}{2}\left(1-F_{si}\right)\sigma_{s}^{2}+\frac{1}{2}\left(1-F_{di}\right)\sigma_{d}^{2}+\sigma_{e}^{2}}{\sigma_{e}^{2}}\right]}^{-1}.$$

Random genetic effects and residuals have the assumed covariance:

$$\mathrm{Var}[\begin{array}{c} a_{s}\\ a_{d}\\ \varepsilon \end{array}]=\left[\begin{array}{ccc} \bar{G}\sigma_{s}^{2} & \bar{G}\sigma_{sd} & 0\\ \bar{G}\sigma_{sd} & \bar{G}\sigma_{d}^{2} & 0\\ 0 & 0 & W\sigma_{e}^{2} \end{array}\right].$$

The resulting mixed model equations are:

$$\left[\begin{array}{ccc} X\prime W^{-1}X & X\prime W^{-1}Z_{s} & X\prime W^{-1}Z_{d}\\ Z\prime_{s}W^{-1}X & Z\prime_{s}W^{-1}Z_{s}+{\bar{G}}^{-1}\alpha_{1} & Z\prime_{s}W^{-1}Z_{d}+{\bar{G}}^{-1}\alpha_{2}\\ Z\prime_{d}W^{-1}X & Z\prime_{d}W^{-1}Z_{s}+{\bar{G}}^{-1}\alpha_{2} & Z\prime_{d}W^{-1}Z_{d}+{\bar{G}}^{-1}\alpha_{3} \end{array}\right][\begin{array}{c} \beta \\ a_{s}\\ a_{d} \end{array}]=\left[\begin{array}{c} X\prime W^{-1}y\\ Z\prime_{s}W^{-1}y\\ Z\prime_{d}W^{-1}y \end{array}\right],$$

with

$$\left[\begin{array}{cc} \alpha_{1} & \alpha_{2}\\ \alpha_{2} & \alpha_{3} \end{array}\right]=\sigma_{e}^{2}{\left[\begin{array}{cc} \sigma_{s}^{2} & \sigma_{sd}\\ \sigma_{sd} & \sigma_{d}^{2} \end{array}\right]}^{-1}.$$

The general model allows for any combination of observation equations (1) to (5) to provide a large degree of flexibility in parent-of-origin analyses. One may choose a model variant to minimize the number of equations for random genetic effects by using as many reduced observation equations as possible, but at the cost of recomputing the weights when estimating the components of variance. Alternatively, one may avoid the repeated recomputation of weights by representing all individuals with an observation using gametic effects. The underlying reason for this flexibility is that for the given data (observations, fixed effects, and pedigree), each possible general imprinting model has as an equivalent unique classical gametic model (as shown in Appendices A2 and A3). Consequently, any two general models that share the same equivalent classical model are also equivalent, and can replace each other, especially when estimating the components of variance.

#### Direct inversion of the generalized gametic relationship matrix:

Setting up the inverse generalized gametic relationship matrix is crucial for any large-scale application. One can derive rules for direct inversion by factoring the inverse ${\bar{G}}^{-1}$ into inverses of a matrix $T^{\prime }$ and a diagonal matrix D of inverse Mendelian sampling variances:

$${\bar{G}}^{-1}={\left(T^{\prime }\right)}^{-1}D^{-1}T^{-1}.$$

This is a known principle based on the direct inversion of the numerator relationship matrix (Henderson 1976; Quaas 1976) and the classical gametic relationship matrix (Schaeffer *et al.* 1989). The matrix ${\left(T^{\prime }\right)}^{-1}$ is lower triangular, as shown in Supplementary Figure S2.1. The underlying pedigree for this example (see Supplementary Part S2) comprises 12 individuals. Single transmitting abilities represent nine individuals and a pair of two gametic effects denotes each of the remaining three. The last column of the pedigree file indicates these two alternative types of representations by values of one and two, respectively. Consequently, the dimensions of the inverse of the example are 15 × 15 and each of the 15 rows of ${\left(T^{\prime }\right)}^{-1}$pertains to one of these effects. However, for the direct inversion, it is necessary to assess the types of genetic effects for all individuals and also for their parents. An individual’s transmitting ability may be derived from two unknown parents (a-00) or a single unknown parent, where the known parent may be represented by a transmitting ability (a-0a, a-a0) or two gametic effects (a-0gg, a-gg0). Two known parents may be represented as any combination of transmitting abilities or gametic effects (a-aa, a-agg, a-gga, a-gggg). Similarly, a gametic effect may be derived from an unknown parent (g-0), or a known parent denoted by either a transmitting ability or two gametic effects (g-a, g-gg). These 12 cases need to be distinguished for the direct inversion of the generalized gametic relationship matrix. Each of these cases appears at least once in the example pedigree. The last column of Supplementary Figure S2.1 indicates the respective case for each effect that relates to a particular row of the lower-triangular matrix. It should be noted that the six cases comprising a-0gg, a-gg0, a-agg, a-gga, a-gggg, and g-a are specific to the generalized gametic relationship matrix because they do not appear in the direct inversion of the numerator relationship matrix (involving only a-00, a-0a, a-a0, and a-aa) or the classical gametic relationship matrix, where only g-0 and g-gg need to be distinguished.

The Mendelian sampling variances that define the diagonal elements of D are different for the transmitting abilities and gametic effects. Furthermore, they depend on the occurrence of unknown parents and the inbreeding coefficients of the known ones. In particular, we have $F_{\text{known}\;\text{parent}}$ when an individual with a transmitting ability in the matrix has only one known parent, or $F_{\text{sire}}$ and $F_{\mathrm{dam}}$ in the case of full parentage information. For gametes, we need to account for the inbreeding coefficient $F_{\text{parent}}$ of the known parent from which a gamete is derived. Accordingly, the 12 cases (a-00, a-0a, …, g-00) are grouped into the following five classes with distinct formulae for the inverse Mendelian sampling variance δ:

$$\begin{array}{ll} a-00 & \delta =2\\ a-0a,\;a-a0,\;a-0\text{gg},\;a-\text{gg}0 & \delta ={\left[\frac{1}{2}\left(1-F_{\mathrm{known}\;\mathrm{parent}}\right)\right]}^{-1}\\ a-\text{aa},\;a-\text{agg},\;a-\text{gga},\;a-\text{gggg} & \delta =2{\left(\frac{1}{2}\left[\frac{1}{2}\left(1-F_{\mathrm{sire}}\right)+\frac{1}{2}\left(1-F_{\mathrm{dam}}\right)\right]\right)}^{-1}\\ g-a,\;g-\text{gg} & \delta =2{\left[\frac{1}{2}\left(1-F_{\mathrm{parent}}\right)\right]}^{-1}\\ g-0 & \delta =1 \end{array}.$$

It is possible to construct the inverse generalized gametic relationship matrix from the pedigree in a step-by-step manner for any arbitrary order of genetic effects. In each step, a matrix contribution $U_{i}$ is added for genetic effect i to a matrix comprising an inverse ${\bar{G}}_{i-1}^{-1}$ that already includes the preceding i−1 effects and zeroes:

$${\bar{G}}_{i}^{-1}=\left[\begin{array}{cc} {\bar{G}}_{i-1}^{-1} & 0\\ 0^{\prime } & 0 \end{array}\right]+U_{i},$$

where 0 is a column vector of i−1 zeros and:

$$U_{i}=u_{i}{u^{\prime }}_{i}\delta_{i}$$

is the contribution made for each genetic effect i. The row vector ${u^{\prime }}_{i}$comprises all zeros, except for the elements that correspond to the genetic effects of the respective parent(s). The *i*-th element (equal to unity) at least is a nonzero in this vector. If present, all other nonzero elements are negative, with values of either $-\frac{1}{2}$ or $-\frac{1}{4}$. Thus, the number of nonzero entries varies from one to five, which can be derived from the rows in the example triangular matrix ${\left(T^{\prime }\right)}^{-1}$ in Supplementary Figure S2.1. Table 1 summarizes the nonzero coefficients in ${u^{\prime }}_{i}$ and their indices for all 12 possible cases. The nonzero elements of the resulting matrix $U_{i}=u_{i}{u^{\prime }}_{i}\delta_{i}$ correspond to the (scaled) cross-products of the elements of the nonzero vector, and their coordinates in the matrix are the respective combinations of indices.

**Table 1:** Size and indices of nonzero elements of vectors $u\prime_{i}$ by type of genetic effect (a: transmitting ability; g: gametic effect)

Type of effect	Case	Nonzero elements in $u\prime_{i}$	Indices of nonzero elements
a	a-00	1	i
a	a-0a	$\begin{array}{cc} -\frac{1}{2} & 1 \end{array}$	d, i
a	a-0gg	$\begin{array}{ccc} -\frac{1}{4} & -\frac{1}{4} & 1 \end{array}$	u, v, i
a	a-a0	$\begin{array}{cc} -\frac{1}{2} & 1 \end{array}$	s, i
a	a-gg0	$\begin{array}{ccc} -\frac{1}{4} & -\frac{1}{4} & 1 \end{array}$	p, q, i
a	a-aa	$\begin{array}{ccc} -\frac{1}{2} & -\frac{1}{2} & 1 \end{array}$	s, d, i
a	a-agg	$\begin{array}{cccc} -\frac{1}{2} & -\frac{1}{4} & -\frac{1}{4} & 1 \end{array}$	s, u, v, i
a	a-gga	$\begin{array}{cccc} -\frac{1}{4} & -\frac{1}{4} & -\frac{1}{2} & 1 \end{array}$	p, q, d, i
a	a-gggg	$\begin{array}{ccccc} -\frac{1}{4} & -\frac{1}{4} & -\frac{1}{4} & -\frac{1}{4} & 1 \end{array}$	p, q, u, v, i
g	g-a	$\begin{array}{cc} -1 & 1 \end{array}$	s, i
g	g-gg	$\begin{array}{ccc} -\frac{1}{2} & -\frac{1}{2} & 1 \end{array}$	p, q, i
g	g-0	1	I

The cases indicate unique combinations of types of genetic effects in an individual and its parents. The indices comprise: i: number of genetic effects; d: transmission ability of dam; s: transmission ability of sire; u: paternal gamete of dam; v: maternal gamete of dam; p: paternal gamete of sire; and q: maternal gamete of sire. For gametic effects (cases g-a and g-gg), an individual’s paternal gamete is assumed. For a maternal gamete, the indices of the genetic effects of the respective effects of the known parent are indexed in the same manner as for a sire.

#### Transforming measured genotypes in a generalized genomic gametic relationship matrix:

Parent-of-origin analyses may also use genomic relationships, or combined genomic and pedigree relationships. However, a specific feature of this approach is that ordinary marker genotypes (AA, AB, BB) are not sufficient. Instead the parental origins of the marker alleles at each locus must be inferred (Lawson *et al.* 2013, and references therein) and summarized as ordered genotypes of AA, AB, BA, and BB, where the first allele is paternally derived, but this is not always possible for all members of a genealogy. In this case, the principles described above are beneficial for integrating ordered and unordered genomic information into a single genomic version of the generalized gametic relationship matrix.

Let us assume that all tindividuals are genotyped with p markers and all genotypes phased into 2t haplotypes. Information regarding the number (zero or one at each locus before centering) of minor alleles for all marker loci on each haplotype can be summarized in a 2t×p matrix C, which is mean-centered column by column. For this matrix, each individual i contributes two p-row vectors ${c^{\prime }}_{i1}$ and ${c^{\prime }}_{i2}$, with centered allele indicators for its first and second haplotypes. Matrix C can then be split into two submatrices $C_{v}$ and $C_{u}$:

$$C=\left[\begin{array}{c} C_{v}\\ C_{u} \end{array}\right].$$

For imprinting analyses, at least all u individuals with phenotypes need to have paternal and maternal haplotypes identified in $C_{u}$. Thus, we must add at least one preceding generation without records but with genotypes. All haplotypes in matrix partition $C_{v}$ are unordered in the case of a single generation. If the additional v genotyped individuals contain more than a single successive generation, then only part of their genotypes may qualify as ordered where the exceptions come from the founders.

A genomic gametic relationship matrix can be derived from C:

$$G_{g}=\frac{CC^{\prime }}{s}=\left[\begin{array}{cc} C_{u}{C^{\prime }}_{u} & C_{u}{C^{\prime }}_{v}\\ C_{v}{C^{\prime }}_{u} & C_{v}{C^{\prime }}_{v} \end{array}\right]\frac{1}{s}=\left[\begin{array}{cc} G_{\mathrm{guu}} & G_{\mathrm{guv}}\\ G_{\mathrm{gvu}} & G_{\mathrm{gvv}} \end{array}\right],$$

where s is a scaling factor, $s=\sum p_{j}\left(1-p_{j}\right)$, and $p_{j}$ is the allele frequency at marker j.

In all cases where the parental origin of the two haplotypes can be traced back, the first haplotype of each individual is assumed to be paternal and the second maternal (${c^{\prime }}_{i1}={c^{\prime }}_{ip}$and ${c^{\prime }}_{i2}={c^{\prime }}_{im}$); otherwise, the ordering of haplotypes is arbitrary. We apply the concept of generalization described above. One can define a transformation matrix $K^{\prime }$ such that for all individuals i with unordered genomic information, the two row vectors ${c^{\prime }}_{i1}$ and ${c^{\prime }}_{i2}$ are replaced by their averages:

$${\bar{c}}_{i}=\frac{1}{2}\left(c_{i1}+c_{i2}\right).$$

The vector ${\bar{c}}_{i}$ does not depend on the order or the parental origin of the haplotypes of an individual:

$${\bar{c}}_{i}=\frac{1}{2}\left(c_{i1}+c_{i2}\right)=\frac{1}{2}\left(c_{ip}+c_{im}\right),$$

*i.e.*, ${\bar{c}}_{i}$ is also the vector of average centered paternal and maternal allele indicators. Consequently, a generalized genomic gametic relationship matrix can be defined as:

$${\bar{G}}_{g}=K^{\prime }CC^{\prime }K\frac{1}{s}=K^{\prime }G_{g}K,$$

where $K^{\prime }$ is defined as above. The partition $C_{u}{C^{\prime }}_{u}$ of $G_{g}$ can be used to determine only the ordered genomic information of all individuals with phenotypes, and thus, it is sufficient to estimate the components of genetic variance in a parent-of-origin analysis. It is possible to estimate all of the respective gametic effects of these individuals. The entire matrix $G_{g}$ represents the gametic effects (as sire and dam) for all individuals, including those with no phenotypes. By design, the generalized variant ${\bar{G}}_{g}$ is also appropriate for parent-of-origin analyses and there are no other requirements for $K^{\prime }$ in the same manner as the pedigree-derived counterpart. Thus, the general model for parent-of-origin analyses is also applicable to genomic relationships provided that the marker haplotypes of individuals with observations are ordered.

### Example data in mice

The mouse line DUKs was maintained for 153 generations as a control for a long-term growth selection experiment (Bünger *et al.* 1998) without any intentional selection conducted at the Leibniz-Institute for Farm Animal Biology. Young DUKs mice were weaned at 21 days of age, and up to five males and four females from each litter were then further reared in two separate cages. At an age of 42 days, the body mass (BM42) was measured for two randomly selected males (BM42 varied from 9.33 g to 41.73 g, with an average of 29.38 g). Randomly selected males and females from about 50% of all litters formed each next generation. This percentage varied from generation to generation due to random fluctuations in the pregnancy rate. Males with a record for BM42 generally did not become a sire, but there were some exceptions when excessively few other males were left in a litter. Therefore, the vast majority (97.67%) of all 13,077 observations were obtained from final progeny. The pedigree size, including all animals with phenotypes and their ancestors was 28,150 animals. Founders were assigned to generation zero. Inbreeding increased up to an average inbreeding coefficient of 0.62 in the last generation, with an average inbreeding coefficient of 0.40 over all generations. In total, 110 generations were included in data analyses as fixed effects (because body weight was not recorded in every generation) as well as 6648 uncorrelated random litter effects. The genetic part of the model was alternatively covered by: (1) breeding values in an *animal model* (AM); (2) a classical *gametic model* (ICM); (3) a *generalized gametic model* (IGM); and (4) a *reduced version* (IRM) of the IGM. The IGM comprised gametic effects only for animals with phenotypes, and transmitting abilities for all others. For the IRM, the underlying pedigree did not include all phenotyped animals without offspring (12,772 in number), thereby leaving only phenotyped parents (305 animals) and their ancestors (15,378 animals). The imprinting models ICM, IGM, and IRM considered genomic imprinting by design, and MCM, MGM, and MRM, respectively, represented the null hypothesis of purely Mendelian inheritance. Furthermore, additional maternal gametic effects augmented the gametic models ICM and IGM to separate them from possible imprinting effects. Inverse relationships of all types were computed using our own Fortran program and REML estimates of variance components were obtained with the software packages ASReml version 4.1 (Gilmour *et al.* 2015) and Echidna version 1.32 (Gilmour 2018). The R-packages “pedigree” version 1.4 (Coster 2013) and “readxl” version 1.3.1 (Wickham *et al.* 2019) in R version 4.0.0 were used to prepare the data. All data, command files, and output files are stored in the RADAR repository. The significance of the imprinting variance was tested by comparing the REML log-likelihood of each imprinting model (ICM, IGM, and IRM) with the REML log-likelihood outcome of the corresponding Mendelian model (MCM, MGM, and MRM). An approximate RLRT with two degrees of freedom was performed (Neugebauer *et al.* 2010a, 2010b). The existence of maternal genetic variance was tested by comparing the REML log-likelihood of an AM without maternal effect with the REML log-likelihood of an AM with maternal effect. Detailed descriptions of the data, methods, and results are presented in the Supplementary Part S3.

### Software and data availability

A collection of six worked toy examples is provided (Part S1 of the Supplementary material provided at https://doi.org/10.25387/g3.14046479) to illustrate the various gametic models with generalized gametic relationships. For each example, the R-code is explicitly presented and used to solve the corresponding mixed model equations. The same examples are part of the detailed *Guide to Practical Implementation*. We also demonstrate in detail how to implement the various mixed models with generalized gametic relationships using the ASReml package. The *Guide to Practical Implementation* is available via the RADAR repository (https://www.doi.org/10.22000/284) and it also includes the source code of a program for directly setting up the inverse of the generalized gametic relationship matrix based on a pedigree file, a detailed program description, and example input and output files.

## Results

When nonimprinted Mendelian inheritance was assumed in our mouse example (see Table 2, upper-left part), the classical *gametic model* (MCM) used 56,300 gametic effects (100%) and the equivalent animal model with a numerator relationship (AM) employed exactly half of that number (28,150; 50%). The *generalized gametic model* with two gametic effects per mouse with record and a single transmitting ability for all others (MGM) was almost exactly in between with 41,227 equations (73%). The number of equations decreased to only 15,683, *i.e.*, 28% of the benchmark, when all final progeny with trait values were represented through the transmitting abilities of their parents (MRM). The corresponding numbers of lower triangle nonzero elements in the inverse were 184,046 (100%) for the MCM and 92,023 (50%) for the AM. The MGM had 103,125 nonzero elements in the lower triangle, which was close to the AM (56%). Only half of that amount was needed by the model with reduced observation equations (MRM), with only 51,372 nonzero elements (28%). When the model allowed for POEs, the absolute number of equations doubled for each model variant (Table 2, upper-right part), and this also applied to the number of saved equations, but their share was the same as that with simple Mendelian inheritance.

**Table 2:** Logarithmic values of the REML (*LogL*), overall number of random genetic effects (no. equa.), and total number of nonzero elements (nonzero) in the lower triangle of the inverted variance–covariance matrix of random genetic effects

	Nonimprinted Mendelian inheritance				Imprinted inheritance		
	AM	MCM	MGM	MRM	ICM	IGM	IRM
***LogL***	–17,891.870	–17,891.870	–17,891.870	–17,891.870^*a*^	–17,868.840	–17,868.840	–17,868.840^*a*^
				–17,894.640^*b*^			–17,875.270^*b*^
**no. equa.**	28,150	56,300	41,227	15,683	112,600	82,454	31,366
**non zero**	92,023	184,046	103,125	51,372	184,046	103,125	51,372
***h* ^2^**	0.497 (±0.030)	0.498 (±0.030)	0.498 (±0.030)	0.565 (±0.025)^*b*^	0.558 (±0.033)	0.558 (±0.033)	0.666 (±0.025)^*b*^
***c* ^2^**	0.275 (±0.015)	0.275 (±0.015)	0.275 (±0.015)	0.248 (±0.019)^*b*^	0.242 (±0.017)	0.242 (±0.017)	0.196 (±0.020)^*b*^
***i* ^2^**	—	—	—	—	0.314 (±0.094)	0.314 (±0.094)	0.391 (±0.083)^*b*^
***r***	—	—	—	—	0.819 (±0.123)	0.819 (±0.123)	0.713 (±0.102)^*b*^
***mat. effect***							
***LogL***	–17,857.280	–17,857.280	–17,857.280	—	–17,855.960	–17,855.960	—
***m* ^2^**	0.127 (±0.034)	0.127 (±0.034)	0.127 (±0.034)	—	0.133 (±0.002)	0.133 (±0.002)	—
***h* ^2^**	0.275 (±0.047)	0.275 (±0.047)	0.275 (±0.047)	—	0.196 (±0.002)	0.196 (±0.002)	—
***c* ^2^**	0.206 (±0.016)	0.206 (±0.016)	0.206 (±0.016)	—	0.188 (±0.009)	0.188 (±0.009)	—
***i* ^2^**	—	—	—	—	0.256 (not es.)	0.256 (not es.)	—
***r***	—	—	—	—	0.907 (not es.)	0.907 (not es.)	—

Heritability (*h*^2^), relative litter variance (*c*^2^), relative imprinting variance (*i*^2^), and correlations between gametic parental effects (*r*) are provided with their standard errors in brackets. *LogL*, relative maternal genetic variance (*m*^2^), *h*^2^, and *c*^2^ were also estimated with a model that included a maternal genetic effect (mat. effect). AM, animal model; MCM, classical gametic model that assumes nonimprinted Mendelian inheritance; MGM, generalized gametic model that assumes nonimprinted Mendelian inheritance; MRM, generalized reduced gametic model that assumes nonimprinted Mendelian inheritance; ICM, imprinting model with classical gametic relationships; IGM, imprinting model with generalized gametic relationships; IRM, imprinting model with generalized reduced gametic relationships.

a The number of iterations was fixed to one and weightings were calculated based on variance component estimates from the corresponding classical models (MCM and ICM).

b Multiple runs were conducted with adapted weightings until *LogL* stabilized. Multiple iterations were allowed per run in order to reach convergence.

Not es. = standard error could not be estimated.

It should be noted that the REML log-likelihood outcomes and the genetic parameters were equal for the Mendelian models and for the imprinting models. The reduced model versions (MRM, IRM) yielded the same REML log-likelihood as the gametic and generalized gametic versions, but with only a single iteration and identical (co-)variance parameters. The results differed when analyses with adapted weightings were repeated until the REML log-likelihood outcomes stabilized (Table 2).

**Figure 1:**
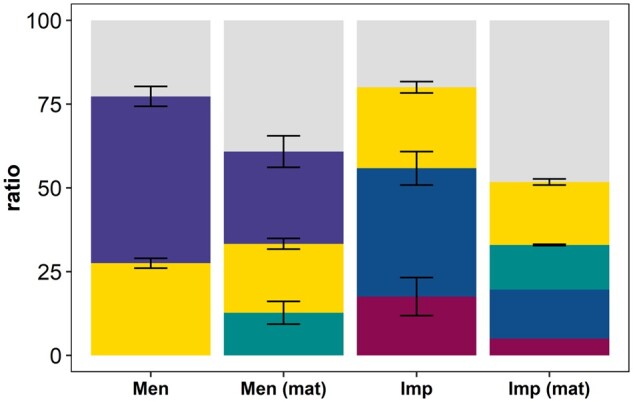
Phenotypic variance of body mass measured in the DUKs mouse line partitioned into the residual variance (gray), additive genetic variance (purple), litter variance (yellow), maternal genetic variance (green), Mendelian variance (blue), and imprinting variance (red). The Mendelian variance was derived by subtracting the imprinting variance from the (direct) additive genetic variance. The variance components were estimated using a model assuming pure Mendelian inheritance (Men), a model assuming Mendelian inheritance and maternal genetic effects [Men (mat)], a model assuming the existence of genomic imprinting but excluding maternal genetic effects (Imp), and a full model that also includes maternal genetic effects [Imp (mat)]. Error bars indicate standard errors. For the Imp (mat) model, the standard errors could not be estimated for all components.

Excluding maternal effects, the imprinting analyses yielded significant imprinting variances with an RLRT of 46.06 (*P *=* *9.96 × 10^−11^ with DF = 2) for the ICM and IGM, and an RLRT of 38.74 (*P *=* *3.87 × 10^−9^ with DF = 2) for the IRM. Using the ICM and IGM, POEs explained 31.40% (±9.40%) of the total genetic variance and 17.52% (±5.68%) of the phenotypic variance (Figure 1). By applying the IRM, POEs explained 39.10% (±8.30%) of the total genetic variance (Table 2). Heritability estimates ranged from 55.80% (±3.30%; ICM and IGM) to 66.60% (±2.50%; IRM). All of the estimated genetic parameters are shown in Table 2. Estimates of the variance and covariance components are summarized in Supplementary Table S3.2. Supplementary Figure S3.3 shows that there was only slight genetic progress in BM42 with about 5 g in 146 generations.

Neglecting imprinting effects (Mendelian inheritance), the maternal genetic variance was significant with an RLRT of 69.18 (*P *=* *8.99 × 10^−17^ with DF = 1). Maternal genetic effects explained 12.70% (±3.40%) of the phenotypic variation (Table 2; Figure 1). Direct heritability decreased from 49.80% (±3.00%) to 27.50% (±4.70%) when maternal effects were included (Figure 1).

Exceptionally slow convergence was observed when both maternal and imprinting effects were included in the model. The log-likelihood improved little compared with the Mendelian model that included a maternal genetic effect (Table 2), so the imprinting variance was considered to be not significant (RLRT = 2.64; *P *=* *0.45 with DF = 3). By contrast, the maternal genetic variance was found to be significant compared with an imprinting model without maternal genetic effects (RLRT = 25.76; *P *=* *1.07 × 10^−5^ with DF = 3). The direct heritability (19.60%; ±0.20%) and relative litter variance (18.80%; ±0.90%) decreased further when all types of POEs were considered, and an increase in relative maternal variance (13.30%; ±0.20%) was observed (Table 2; Figure 1).

## Discussion

The outlined generalization introduces elements of the *reduced imprinting model* into the *gametic model*, and vice versa, to obtain increased flexibility and substantial reductions in terms of the number of equations. The latter is especially important for estimating the components of variance (Shor *et al.* 2019). The matrix $\bar{G}$ contains two limiting cases that set the boundaries for the ratio of equations that can possibly be eliminated. The first is the classical gametic relationship matrix itself (dimensions of 2*t* × 2*t*) when $K^{\prime }$ is an identity matrix. The other limiting case is $K^{\prime }=I⊗\left[\begin{array}{cc} \frac{1}{2} & \frac{1}{2} \end{array}\right]$, such that $\bar{G}=\frac{1}{2}A$ with dimensions of *t *×* t*. Therefore, the reduction in the number of equations for genetic effects can range from 0% to 50% compared with a classical *gametic model*. However, the actual reduction depends on the specific details of each data set. Furthermore, a specific fraction of individuals with records (*i.e.*, with phenotypes) might have not reproduced at all or not yet reproduced at the time of data recall, *i.e.*, they appear as final progeny, which allows them to be represented by reduced observational equations rather than having their own gametic effects in the model. This may result in a reduction of even more than 50% of the full set of gametic equations.

In general, sex-specific traits such as the litter size, number of eggs, or milk yield allow all males to be represented by their transmitting abilities. Thus, the resulting number of equations is considerably smaller compared with a trait commonly recorded in both sexes. The family structure also has an effect, where it is possible to save more equations with smaller groups of paternal offspring if sires without their phenotype have transmitting abilities as genetic effects. Furthermore, in imprinting analyses, it is reasonable to add a high ratio of ancestors without phenotypes to better reflect inbreeding and the relationships between genetic effects as sire and dam. Thus, we can minimize the burden of additional equations by estimating the transmitting abilities of these ancestors rather than their gametic effects.

In certain cases, we could refrain from using reduced observational equations, which have the advantage that no weights are required that depend on currently undetermined components of the variance. This approach may be beneficial in Bayesian approaches that employ Markov Chain Monte Carlo methods, where the values of the components of variance change from iteration to iteration. By exploiting the flexibility of the generalized approach, weights become obsolete by representing all individuals with phenotypes by two gametic effects, regardless of whether they are final progeny. This helps to offset the computational burden due to repeated reweighting. In addition, we can integrate individuals without observations by single equations. In previous REML estimations of the components of variance with reduced imprinting models (Neugebauer *et al.* 2010a, 2010b; Blunk *et al.* 2017a, 2017b), equal weights were used at the start and later recalculated repeatedly until final convergence was reached. For the mouse example data, this led to an REML log-likelihood that was slightly below the actual maximum (Table 2) under both Mendelian (MRM: –17,894.64 *vs* –17,891.87) and imprinted inheritance (IRM: –17,875.27 *vs* –17,868.84). No similar behavior was detected in the cited livestock studies, which may be explained by the much higher degree of inbreeding in the mouse line (up to about 60%).

Excluding this difficulty finding the maximum, all of the REML log-likelihood values were identical (Table 2) if we assumed the same underlying genetic model, irrespective of the equivalent representations employed for the gametic relationships. The same was clearly true for all of the estimated variance components (Supplementary Table S3.2). Thus, our mouse example data analyses technically reproduced all of the theoretically derived model equivalences.

Among the DUKs mouse data, none of the dams had a trait value, which can hinder the estimation of variance components in the presence of maternal genetic effects (Heydarpour *et al.* 2008), and it probably explains the observed slow convergence rate for the full model with three genetic effects. An imprinting model variant with reduced observation equations (IRM) including maternal genetic effects was not applied to the mouse data set because the *reduced imprinting model* cannot separate the imprinting variance from maternal genetic variance components when both are present, as mentioned in the introduction and proven in Appendix A4. A solution to this issue involves avoiding reduced equations and explicitly representing individuals with phenotypes based on their gametic effects in a model that also includes maternal genetic effects. In principle, it should then be possible to separate the gametic variances as sire and dam from the maternal genetic variance (Appendix A5). However, in practice, limitations in the amount and structure of the data may hinder this approach, as also observed in a human genetic epidemiological study (Blunk *et al.* 2020) and for Mendelian models (Heydarpour *et al.* 2008). Similar to maternal effects models, other types of imprinting models may also include more than a single genetic effect as sire and dam per individual, *e.g.*, random regression models or multitrait models. However, they are all hindered by a large number of gametic equations, and thus they benefit even more from generalized relationships.

No significant imprinting effects were found to affect the variations in body mass in the DUKs mouse line, although imprinting is known to have important functions in stem cells, neuronal differentiation, and growth (Plasschaert and Bartolomei 2014). By contrast, maternal effects seemed to play an important role, which was expected because maternal genetic effects on body mass traits in mice have been known for a long time (Hanrahan and Eisen 1973). When maternal effects were neglected in the analysis, direct heritability seemed to be overestimated, as shown in Figure 1 for the Mendelian and the imprinting models. Furthermore, when maternal effects were not considered, the imprinting variance was inflated and the estimates were reduced when maternal effects were included in the model (Figure 1). The litter variance seemed to be largely unaffected by the inclusion of POEs and it explained about a quarter of the phenotypic variance (21–27%).

In applications where all v individuals with phenotypes plus at least one preceding generation have measured genotypes and the variance components need be estimated, it is sufficient to include only the subset of these v individuals with their genomic covariance $G_{\mathrm{gvv}}$. If the genetic effects of the u founders as sire and dam are of interest, then either $G_{g}$ or ${\bar{G}}_{g}$ is selected. An example is an F_2_ line-cross experiment with phenotypes recorded only in the F_2_ generation, where the genotypes of the F_1_ and P_0_ generations are only required for phasing and determining the line origins of the markers.

To assess genomic imprinting effects, a two-step approach was developed for the *reduced imprinting model*. In the first step, imprinting effects (differences between transmitting abilities as sires and dams) must be estimated for parents in pedigree-based analyses. After de-regressing these estimates and using the corresponding reliabilities, they can be employed as dependent variables in a genome-wide association study (Blunk *et al.* 2019) where the marker genotypes of the parents can be unordered. Further investigations are required to determine whether we can extend this two-step approach to generalized gametic relationships, which would require de-regressing the differences between genetic effects of all types (both gametic effects and transmitting abilities as sire and dam) in a similar manner, before they are subjected to a genome scan.

Animal breeders frequently combine large pedigrees comprising smaller cohorts of genotyped individuals. Certain individuals are then the first to be genotyped in their genealogy and one can trace their pedigree further back. In contrast to their own descendants, it is not possible to order the haplotypes of these candidates, and thus, it is uncertain whether the first of two unordered marker haplotypes matches the paternal gametic effect in a pedigree-derived gametic relationship matrix or the maternal one. Consequently, a combined relationship matrix cannot be constructed that is suitable for parent-of-origin analyses. We can solve this problem by collapsing gametic effects into transmitting abilities in the genomic relationships as well as in the pedigree-derived relationships. The generalized pedigree relationships for all animals can then be combined with their matching generalized genomic counterparts ${\bar{G}}_{g}$ for the genotyped cohort in a manner that facilitates integration of unordered genomic information. The available theory (Legarra *et al.* 2009; Christensen and Lund 2010; Aguilar *et al.* 2010) can be used to combine pedigree-derived relationships ($\bar{G}$) and genomic relationships (${\bar{G}}_{g}$) into a joint matrix, at least in the many cases where candidates with unordered genotypes have no records, such as dairy bulls.

In conclusion, the generalized gametic relationship matrix provides the necessary flexibility to adapt imprinting analyses to specific computational and analytical requirements in many situations by using tailored versions of the general imprinting model. The most important features of this method are the effective estimation of the imprinting variance in REML and Bayesian approaches in case where the parents have records, the inclusion of maternal genetic effects, and genomic relationships that integrate ordered and unordered genomic information. Overall, these new options are expected to stimulate systematic research into the importance of POEs for the genetic variation in quantitative traits in farm animals and other species.

## Appendix

### Appendix A1: Equivalence of the classical gametic model and generalized gametic model where all individuals with phenotypes have two gametic effects

Both models have the same expectation E(y)=Xβ for the vector of observations y. The variance of observations in the classical gametic model is $\mathrm{Var}\left(y\right)=Q_{c}+I\sigma_{e}^{2}$, where

$$\begin{array}{c} Q_{c}=\left[\begin{array}{cc} Z_{s} & Z_{d} \end{array}\right]\left[\begin{array}{cc} G\sigma_{s}^{2} & G\sigma_{sd}\\ G\sigma_{sd} & G\sigma_{d}^{2} \end{array}\right]\left[\begin{array}{c} {Z^{\prime }}_{s}\\ {Z^{\prime }}_{d} \end{array}\right]\\ =Z_{s}GZ_{s}{}^{\prime }\sigma_{s}^{2}+Z_{s}GZ_{d}{}^{\prime }\sigma_{sd}+Z_{d}GZ_{s}{}^{\prime }\sigma_{sd}+Z_{d}GZ_{d}{}^{\prime }\sigma_{d}^{2} \end{array}.$$

The first term can be rewritten as

$$Z_{s}GZ_{s}{}^{\prime }=\left[\begin{array}{cc} 0^{2u} & Z_{s}^{v} \end{array}\right]\left[\begin{array}{cc} G_{uu} & G_{uv}\\ G_{uv}{}^{\prime } & G_{vv} \end{array}\right]\left[\begin{array}{c} {\left(0^{2u}\right)}^{\prime }\\ {\left(Z_{s}^{v}\right)}^{\prime } \end{array}\right]=Z_{s}^{v}G_{vv}Z_{s}^{v}{}^{\prime }.$$

Likewise,

$$Z_{s}GZ_{d}{}^{\prime }=Z_{s}^{v}G_{vv}Z_{d}^{v}{}^{\prime },\;\;Z_{d}GZ_{s}{}^{\prime }=Z_{d}^{v}G_{vv}Z_{s}^{v}{}^{\prime }\;\;\text{and}\;Z_{d}GZ_{d}{}^{\prime }=Z_{d}^{v}G_{vv}Z_{d}^{v}{}^{\prime }.$$

Finally,

$$Q_{c}=Z_{s}^{v}G_{vv}Z_{s}^{v}{}^{\prime }\sigma_{s}^{2}+Z_{s}^{v}G_{vv}Z_{d}^{v}{}^{\prime }\sigma_{sd}+Z_{d}^{v}G_{vv}Z_{s}^{v}{}^{\prime }\sigma_{sd}+Z_{d}^{v}G_{vv}Z_{d}^{v}{}^{\prime }\sigma_{d}^{2}.$$

In the generalized case, the variance of observations is

$$\mathrm{Var}(y)=Q_{g}+I\sigma_{e}^{e}$$

with

$$Q_{g}=\left[\begin{array}{cc} Z_{s}K & Z_{d}K \end{array}\right]\left[\begin{array}{cc} \bar{G}\sigma_{s}^{2} & \bar{G}\sigma_{sd}\\ \bar{G}\sigma_{sd} & \bar{G}\sigma_{d}^{2} \end{array}\right]\left[\begin{array}{c} K^{\prime }{Z^{\prime }}_{s}\\ K^{\prime }{Z^{\prime }}_{d} \end{array}\right].$$

We use $Z_{s}K=\left[\begin{array}{cc} 0^{u} & Z_{s}^{v} \end{array}\right]$ and $Z_{d}K=\left[\begin{array}{cc} 0^{u} & Z_{d}^{v} \end{array}\right]$, and rewrite

$$Z_{s}K\bar{G}K^{\prime }Z_{s}{}^{\prime }=\left[\begin{array}{cc} 0^{u} & Z_{s}^{v} \end{array}\right]\left[\begin{array}{cc} \frac{1}{2}A_{u} & S_{uv}\\ S_{uv}{}^{\prime } & G_{vv} \end{array}\right]\left[\begin{array}{c} {\left(0^{u}\right)}^{\prime }\\ {\left(Z_{s}^{v}\right)}^{\prime } \end{array}\right]=Z_{s}^{v}G_{vv}Z_{s}^{v}{}^{\prime }.$$

In the same manner,

$$Z_{s}K\bar{G}K^{\prime }Z_{d}{}^{\prime }=Z_{s}^{v}G_{vv}Z_{d}^{v}{}^{\prime },\;\;Z_{d}K\bar{G}K^{\prime }Z_{s}{}^{\prime }=Z_{d}^{v}G_{vv}Z_{s}^{v}{}^{\prime },\;\text{and}\;\;Z_{d}K\bar{G}K^{\prime }Z_{d}{}^{\prime }=Z_{d}^{v}G_{vv}Z_{d}^{v}{}^{\prime }.$$

We then obtain:

$$Q_{g}=Z_{s}^{v}G_{vv}Z_{s}^{v}{}^{\prime }\sigma_{s}^{2}+Z_{s}^{v}G_{vv}Z_{d}^{v}{}^{\prime }\sigma_{sd}+Z_{d}^{v}G_{vv}Z_{s}^{v}{}^{\prime }\sigma_{sd}+Z_{d}^{v}G_{vv}Z_{d}^{v}{}^{\prime }\sigma_{d}^{2}=Q_{c}.$$

From $Q_{g}=Q_{c}$, it follows that Var(y) is the same in both models and they are equivalent.

### Appendix A2: Equivalence of classical and generalized gametic relationships in reduced models

We consider a reduced model with classical gametic relationships. With classical gametic relationships, all parents of the final progeny and their ancestors have two gametic effects in the model with covariance G. In the generalized case, the gametic effects of u of them are collapsed into the transmitting abilities, whereas the remaining v individuals retain their gametic effects. For the sake of generality, the latter group among an arbitrary choice of others includes all parents who may have records. Parents with phenotypes need to have gametic effects, whereas all other individuals may be modeled by gametic effects or transmitting abilities. The final progeny have no genetic effects of their own in the reduced model, so the variance of the residuals $W\sigma_{e}^{2}$ is not affected by relationships. With classical gametic relationships, the variance of observations is:

$$\mathrm{Var}\left(y\right)=Q_{c}+W\sigma_{e}^{2},$$

where

$$Q_{c}=\left[\begin{array}{cc} Z_{s} & Z_{d} \end{array}\right]\left[\begin{array}{cc} G\sigma_{s}^{2} & G\sigma_{sd}\\ G\sigma_{sd} & G\sigma_{d}^{2} \end{array}\right]\left[\begin{array}{c} {Z^{\prime }}_{s}\\ {Z^{\prime }}_{d} \end{array}\right].$$

The incidence matrix $Z_{s}$ for genetic effects can be partitioned as $Z_{s}=\left[\begin{array}{cc} Z_{s}^{2u} & Z_{s}^{v} \end{array}\right]$. There are two adjacent columns per individual in the first partition, *i.e.*, $Z_{s}^{2u}=Z_{s}^{u}⊗\left(\begin{array}{cc} \frac{1}{2} & \frac{1}{2} \end{array}\right)=Z_{s}^{u}\left[I_{u}⊗\left(\begin{array}{cc} \frac{1}{2} & \frac{1}{2} \end{array}\right)\right]$, where $Z_{s}^{u}$ is the corresponding partition from the same type of incidence matrix in the model with generalized gametic relationships and $I_{u}$ is a u × u identity matrix. It should be noted that a multiplication with K cannot applied for the conversion of the matrix $\left[\begin{array}{cc} Z_{s}^{2u} & Z_{s}^{v} \end{array}\right]$ into $\left[\begin{array}{cc} Z_{s}^{u} & Z_{s}^{v} \end{array}\right]$ because $Z_{s}^{v}$ may have both entries of single ones for records of parents and of pairs of one half for records from final progeny. This also applies in an analogous manner to $Z_{d}^{2u}$ and $Z_{d}^{u}$.

The first component of $Q_{c}$ is:

$$\begin{array}{c} Z_{s}G{Z^{\prime }}_{s}=\left[\begin{array}{cc} Z_{s}^{2u} & Z_{s}^{v} \end{array}\right]\left[\begin{array}{cc} G_{uu}\sigma_{s}^{2} & G_{uv}\sigma_{s}^{2}\\ G_{uv}\sigma_{s}^{2} & G_{vv}\sigma_{s}^{2} \end{array}\right]\left[\begin{array}{c} {\left(Z_{s}^{2u}\right)}^{\prime }\\ {\left(Z_{s}^{v}\right)}^{\prime } \end{array}\right]=\\ Z_{s}^{2u}G_{uu}{\left(Z_{s}^{2u}\right)}^{\prime }\sigma_{s}^{2}+Z_{s}^{2u}G_{uv}{\left(Z_{s}^{v}\right)}^{\prime }\sigma_{s}^{2}+Z_{s}^{v}{G^{\prime }}_{uv}{\left(Z_{s}^{2u}\right)}^{\prime }\sigma_{s}^{2}+Z_{s}^{v}G_{vv}{\left(Z_{s}^{v}\right)}^{\prime }\sigma_{s}^{2}=\\ Z_{s}^{u}\left[I⊗\left(\begin{array}{cc} \frac{1}{2} & \frac{1}{2} \end{array}\right)\right]G_{uu}{\left[I⊗\left(\begin{array}{cc} \frac{1}{2} & \frac{1}{2} \end{array}\right)\right]}^{\prime }{\left(Z_{s}^{u}\right)}^{\prime }\sigma_{s}^{2}+Z_{s}^{u}\left[I⊗\left(\begin{array}{cc} \frac{1}{2} & \frac{1}{2} \end{array}\right)\right]\\ G_{uv}{\left(Z_{s}^{v}\right)}^{\prime }\sigma_{s}^{2}+Z_{s}^{v}{G^{\prime }}_{uv}{\left[I⊗\left(\begin{array}{cc} \frac{1}{2} & \frac{1}{2} \end{array}\right)\right]}^{\prime }{\left(Z_{s}^{u}\right)}^{\prime }\sigma_{s}^{2}+Z_{s}^{v}G_{vv}{\left(Z_{s}^{v}\right)}^{\prime }\sigma_{s}^{2}\\ =Z_{s}^{u}\frac{1}{2}A{\left(Z_{s}^{u}\right)}^{\prime }\sigma_{s}^{2}+Z_{s}^{u}S_{uv}{\left(Z_{s}^{v}\right)}^{\prime }\sigma_{s}^{2}+Z_{s}^{v}{S^{\prime }}_{uv}{\left(Z_{s}^{u}\right)}^{\prime }\sigma_{s}^{2}+Z_{s}^{v}G_{vv}{\left(Z_{s}^{v}\right)}^{\prime }\sigma_{s}^{2}. \end{array}$$

Similarly,

$$\begin{array}{c} Z_{s}G{Z^{\prime }}_{d}=\left[\begin{array}{cc} Z_{s}^{2u} & Z_{s}^{v} \end{array}\right]\left[\begin{array}{cc} G_{uu}\sigma_{sd} & G_{uv}\sigma_{sd}\\ G_{uv}\sigma_{sd} & G_{vv}\sigma_{sd} \end{array}\right]\left[\begin{array}{c} {\left(Z_{d}^{2u}\right)}^{\prime }\\ {\left(Z_{d}^{v}\right)}^{\prime } \end{array}\right]=\\ Z_{s}^{u}\frac{1}{2}A{\left(Z_{s}^{u}\right)}^{\prime }\sigma_{sd}+Z_{s}^{u}S_{uv}{\left(Z_{s}^{v}\right)}^{\prime }\sigma_{sd}+Z_{s}^{v}{S^{\prime }}_{uv}{\left(Z_{s}^{u}\right)}^{\prime }\sigma_{sd}+Z_{s}^{v}G_{vv}{\left(Z_{s}^{v}\right)}^{\prime }\sigma_{sd} \end{array}$$

and

$$\begin{array}{c} Z_{d}G{Z^{\prime }}_{s}=\left[\begin{array}{cc} Z_{d}^{2u} & Z_{s}^{v} \end{array}\right]\left[\begin{array}{cc} G_{uu}\sigma_{sd} & G_{uv}\sigma_{sd}\\ G_{uv}\sigma_{sd} & G_{vv}\sigma_{sd} \end{array}\right]\left[\begin{array}{c} {\left(Z_{s}^{2u}\right)}^{\prime }\\ {\left(Z_{s}^{v}\right)}^{\prime } \end{array}\right]=\\ Z_{s}^{u}\frac{1}{2}A{\left(Z_{s}^{u}\right)}^{\prime }\sigma_{sd}+Z_{s}^{u}S_{uv}{\left(Z_{s}^{v}\right)}^{\prime }\sigma_{sd}+Z_{s}^{v}{S^{\prime }}_{uv}{\left(Z_{s}^{u}\right)}^{\prime }\sigma_{sd}+Z_{s}^{v}G_{vv}{\left(Z_{s}^{v}\right)}^{\prime }\sigma_{sd} \end{array}$$

and finally,

$$\begin{array}{c} Z_{d}G{Z^{\prime }}_{d}=\left[\begin{array}{cc} Z_{d}^{2u} & Z_{d}^{v} \end{array}\right]\left[\begin{array}{cc} G_{uu}\sigma_{d}^{2} & G_{uv}\sigma_{d}^{2}\\ G_{uv}\sigma_{d}^{2} & G_{vv}\sigma_{d}^{2} \end{array}\right]\left[\begin{array}{c} {\left(Z_{d}^{2u}\right)}^{\prime }\\ {\left(Z_{d}^{v}\right)}^{\prime } \end{array}\right]=\\ Z_{s}^{u}\frac{1}{2}A{\left(Z_{s}^{u}\right)}^{\prime }\sigma_{d}^{2}+Z_{s}^{u}S_{uv}{\left(Z_{s}^{v}\right)}^{\prime }\sigma_{d}^{2}+Z_{s}^{v}{S^{\prime }}_{uv}{\left(Z_{s}^{u}\right)}^{\prime }\sigma_{d}^{2}+Z_{s}^{v}G_{vv}{\left(Z_{s}^{v}\right)}^{\prime }\sigma_{d}^{2} \end{array}.$$

Therefore, $Q_{c}$ can be summarized as:

$$Q_{c}=\left[\begin{array}{cc} \left(\begin{array}{cc} Z_{s}^{u} & Z_{s}^{v} \end{array}\right) & \left(\begin{array}{cc} Z_{d}^{u} & Z_{d}^{v} \end{array}\right) \end{array}\right]\left(\begin{array}{cc} \sigma_{s}^{2} & \sigma_{sd}\\ \sigma_{sd} & \sigma_{s}^{2} \end{array}\right)⊗\left[\begin{array}{cc} \frac{1}{2}A_{uu} & S_{uv}\\ S_{uv} & G_{vv} \end{array}\right]\left[\begin{array}{c} {\left(\begin{array}{cc} Z_{s}^{u} & Z_{s}^{v} \end{array}\right)}^{\prime }\\ {\left(\begin{array}{cc} Z_{d}^{u} & Z_{d}^{v} \end{array}\right)}^{\prime } \end{array}\right],$$

which is equal to the equivalent quantity $Q_{r}$ using generalized gametic relationships:

$$Q_{r}=\left[\begin{array}{cc} \left(\begin{array}{cc} Z_{s}^{u} & Z_{s}^{v} \end{array}\right) & \left(\begin{array}{cc} Z_{d}^{u} & Z_{d}^{v} \end{array}\right) \end{array}\right]\left[\begin{array}{cc} \bar{G}\sigma_{s}^{2} & \bar{G}\sigma_{sd}\\ \bar{G}\sigma_{sd} & \bar{G}\sigma_{d}^{2} \end{array}\right]\left[\begin{array}{c} {\left(\begin{array}{cc} Z_{s}^{u} & Z_{s}^{v} \end{array}\right)}^{\prime }\\ {\left(\begin{array}{cc} Z_{d}^{u} & Z_{d}^{v} \end{array}\right)}^{\prime } \end{array}\right].$$

Thus,

$$\mathrm{Var}\left(y\right)=Q_{c}+W\sigma_{e}^{2}=Q_{r}+W\sigma_{e}^{2},\text{q.e.d}.$$

### Appendix A3: Equivalence between the model with gametic effects for all individuals and the reduced model with gametic effects for parents

We consider a classical gametic model that includes a number f of final progeny. The vector g is partitioned into two components, with the 2f gametic effects of the final f progeny in $g_{f}$ and other gametic effects in $g_{g}$. Then, the covariance of g is:

$$\mathrm{Var}\left(g\right)=\mathrm{Var}\left[\begin{array}{c} g_{g}\\ g_{f} \end{array}\right]=\left[\begin{array}{cc} G_{gg} & G_{gf}\\ {G^{\prime }}_{gf} & G_{ff} \end{array}\right]=G.$$

The incidence matrices for gametic effects are:

$$Z_{s}^{\mathrm{all}}=\left[\begin{array}{cc} Z_{s1}^{g} & 0\\ 0 & Z_{s}^{f} \end{array}\right]\text{and}Z_{d}^{\mathrm{all}}=\left[\begin{array}{cc} Z_{d1}^{g} & 0\\ 0 & Z_{d}^{f} \end{array}\right],$$

where $Z_{s1}^{g}$ ($Z_{d1}^{g}$) relates the observations to the gametic effects as sire (as dam) of individuals that are not in the set of the f final progeny. Accordingly, $Z_{s}^{f}$ ($Z_{d}^{f}$) relates observations of the f final progeny to their respective gametic effects as sire (as dam).

By contrast, in a reduced model, all observations of the f final progeny must be related to the gametic effects as sire (as dam) of their parents. The respective incidence matrices are:

$$Z_{s}^{\mathrm{red}}=\left[\begin{array}{cc} Z_{s1}^{g} & 0\\ Z_{s2}^{g} & 0 \end{array}\right]\text{and}Z_{d}^{\mathrm{red}}=\left[\begin{array}{cc} Z_{d1}^{g} & 0\\ Z_{d2}^{g} & 0 \end{array}\right],$$

where $Z_{s}^{\mathrm{red}}$ and $Z_{d}^{\mathrm{red}}$ have only zero entries in their columns for the gametic effects of final progeny.

The relationships between the incidence matrices of the two types of models are:

$$Z_{s}^{\mathrm{red}}=\left[\begin{array}{cc} Z_{s1}^{g} & 0\\ 0 & Z_{s}^{f} \end{array}\right]-\left[\begin{array}{cc} 0 & 0\\ -Z_{s2}^{g} & Z_{s}^{f} \end{array}\right]=\left[\begin{array}{cc} Z_{s1}^{g} & 0\\ Z_{s2}^{g} & 0 \end{array}\right]=Z_{s}^{\mathrm{all}}-Z_{s}^{\delta }.$$

The matrix $Z_{s}^{\delta }$ is the difference between $Z_{s}^{\mathrm{all}}$ and $Z_{s}^{\mathrm{red}}$:

$Z_{s}^{\delta }=\left[\begin{array}{cc} 0 & 0\\ -Z_{s2}^{g} & Z_{s}^{f} \end{array}\right]$ , and analogously $Z_{d}^{\delta }=\left[\begin{array}{cc} 0 & 0\\ -Z_{d2}^{g} & Z_{d}^{f} \end{array}\right]$,

$$Z_{s}^{\mathrm{all}}=Z_{s}^{\mathrm{red}}+Z_{s}^{\delta },$$

and

$$Z_{d}^{\mathrm{all}}=Z_{d}^{\mathrm{red}}+Z_{d}^{\delta }.$$

To prove the equivalence of the two models, we express the variance of observations Var(y) in terms of model-specific incidence matrices and demonstrate their equality by using the last two identities.

For any reduced observation equation, the variances of the relevant Mendelian sampling effects are part of the residual. For each paternal gamete as sire of a final progeny, the Mendelian sampling effect is the difference between the effect of the paternal gamete and the transmitting ability of the individual’s sire as sire. The respective vector is:

$$m_{s}=Z_{s}^{\delta }g_{f},$$

and the maternal counterpart as dam is:

$$m_{d}=Z_{d}^{\delta }g_{f}.$$

The common covariance matrix is:

$$\mathrm{Var}\left[\begin{array}{c} m_{s}\\ m_{d} \end{array}\right]=[\begin{array}{cc} Z_{s}^{\delta }G{\left(Z_{s}^{\delta }\right)}^{\prime }\sigma_{s}^{2} & Z_{s}^{\delta }G{\left(Z_{d}^{\delta }\right)}^{\prime }\sigma_{sd}\\ Z_{s}^{\delta }G{\left(Z_{d}^{\delta }\right)}^{\prime }\sigma_{sd} & Z_{d}^{\delta }G{\left(Z_{d}^{\delta }\right)}^{\prime }\sigma_{d}^{2} \end{array}]=\left[\begin{array}{cc} Z_{s}^{\delta }G{\left(Z_{s}^{\delta }\right)}^{\prime }\sigma_{s}^{2} & 0\\ 0 & Z_{d}^{\delta }G{\left(Z_{d}^{\delta }\right)}^{\prime }\sigma_{d}^{2} \end{array}\right].$$

The covariance between $m_{s}$ and $m_{d}$ is zero because all rows of $Z_{s}^{\delta }$ have their nonzero entries at places other than the rows of $Z_{d}^{\delta }$, and thus, $Z_{s}^{\delta }G{\left(Z_{d}^{\delta }\right)}^{\prime }$ is a matrix of zeroes.

In particular, the product is:

$$Z_{s}^{\delta }G{\left(Z_{d}^{\delta }\right)}^{\prime }={\left\{\text{sum}\left(z_{d,i}^{\delta }{\left(z_{s,j}^{\delta }\right)}^{\prime }⊙G\right)\right\}}_{ij}={\left\{0\right\}}_{ij},$$

where ⊙ denotes element-wise multiplication, sum () is the sum of all matrix elements in (), and ${\left(z_{d,i}^{\delta }\right)}^{\prime }$ and ${\left(z_{s,j}^{\delta }\right)}^{\prime }$ are the ith and jth rows of the two incidence matrices involved, respectively.

The total Var(y) in the reduced model is:

$$\mathrm{Var}\left(y\right)=Q_{\mathrm{red}}+\mathrm{Var}\left(m_{s}\right)+\mathrm{Var}\left(m_{d}\right)+I\sigma_{e}^{2},$$

with

$$\begin{array}{c} Q_{\mathrm{red}}=\left[\begin{array}{cc} Z_{s}^{\mathrm{red}} & Z_{d}^{\mathrm{red}} \end{array}\right]\left[\begin{array}{cc} \sigma_{s}^{2} & \sigma_{sd}\\ \sigma_{sd} & \sigma_{d}^{2} \end{array}\right]⊗G\left[\begin{array}{c} {\left(Z_{s}^{\mathrm{red}}\right)}^{\prime }\\ {\left(Z_{d}^{\mathrm{red}}\right)}^{\prime } \end{array}\right]\\ =Z_{s}^{\mathrm{red}}G{\left(Z_{s}^{\mathrm{red}}\right)}^{\prime }\sigma_{s}^{2}+Z_{s}^{\mathrm{red}}G{\left(Z_{d}^{\mathrm{red}}\right)}^{\prime }\sigma_{sd}+Z_{d}^{\mathrm{red}}G{\left(Z_{s}^{\mathrm{red}}\right)}^{\prime }\sigma_{sd}+Z_{d}^{\mathrm{red}}G{\left(Z_{d}^{\mathrm{red}}\right)}^{\prime }\sigma_{d}^{2} \end{array}.$$

In the classical gametic model, the variance of observations Var(y) is:

$$Q_{\mathrm{all}}+I\sigma_{e}^{2}.$$

The first component is:

$$\begin{array}{c} Q_{\mathrm{all}}=\left[\begin{array}{cc} Z_{s}^{\mathrm{all}} & Z_{d}^{\mathrm{all}} \end{array}\right]\left[\begin{array}{cc} \sigma_{s}^{2} & \sigma_{sd}\\ \sigma_{sd} & \sigma_{d}^{2} \end{array}\right]⊗G\left[\begin{array}{c} {\left(Z_{s}^{\mathrm{all}}\right)}^{\prime }\\ {\left(Z_{d}^{\mathrm{all}}\right)}^{\prime } \end{array}\right]\\ =\left[\begin{array}{cc} Z_{s}^{\mathrm{red}}+Z_{s}^{\delta } & Z_{d}^{\mathrm{red}}+Z_{d}^{\delta } \end{array}\right]\left[\begin{array}{cc} \sigma_{s}^{2} & \sigma_{sd}\\ \sigma_{sd} & \sigma_{d}^{2} \end{array}\right]⊗G\left[\begin{array}{c} {\left(Z_{s}^{\mathrm{red}}+Z_{s}^{\delta }\right)}^{\prime }\\ {\left(Z_{d}^{\mathrm{red}}+Z_{d}^{\delta }\right)}^{\prime } \end{array}\right] \end{array}.$$

This yields a total of 16 terms, where the first four are:

$$=Z_{s}^{\mathrm{red}}G{\left(Z_{s}^{\mathrm{red}}\right)}^{\prime }\sigma_{s}^{2}+Z_{s}^{\mathrm{red}}G{\left(Z_{d}^{\mathrm{red}}\right)}^{\prime }\sigma_{sd}+Z_{d}^{\mathrm{red}}G{\left(Z_{s}^{\mathrm{red}}\right)}^{\prime }\sigma_{sd}+Z_{d}^{\mathrm{red}}G{\left(Z_{d}^{\mathrm{red}}\right)}^{\prime }\sigma_{d}^{2},$$

and this is equal to $Q_{\mathrm{red}}$ in the reduced model. We also have two more terms:

$$Z_{s}^{\delta }G{\left(Z_{s}^{\delta }\right)}^{\prime }\sigma_{s}^{2}+Z_{d}^{\delta }G{\left(Z_{d}^{\delta }\right)}^{\prime }\sigma_{d}^{2},$$

which are equivalent to $\mathrm{Var}\left(m_{s}\right)+\mathrm{Var}\left(m_{d}\right)$. The remaining 10 terms in:

$$\begin{array}{l} Z_{s}^{\mathrm{red}}G{\left(Z_{s}^{\mathrm{red}}\right)}^{\prime }\sigma_{s}^{2}+Z_{s}^{\delta }G{\left(Z_{s}^{\mathrm{red}}\right)}^{\prime }\sigma_{s}^{2}+\\ Z_{s}^{\mathrm{red}}G{\left(Z_{d}^{\delta }\right)}^{\prime }\sigma_{sd}+Z_{s}^{\delta }G{\left(Z_{d}^{\mathrm{red}}\right)}^{\prime }\sigma_{sd}+Z_{s}^{\delta }G{\left(Z_{d}^{\delta }\right)}^{\prime }\sigma_{sd}+\\ Z_{d}^{\mathrm{red}}G{\left(Z_{s}^{\delta }\right)}^{\prime }\sigma_{sd}+Z_{d}^{\delta }G{\left(Z_{s}^{\mathrm{red}}\right)}^{\prime }\sigma_{sd}+Z_{d}^{\delta }G{\left(Z_{s}^{\delta }\right)}^{\prime }\sigma_{sd}+\\ Z_{d}^{\mathrm{red}}G{\left(Z_{d}^{\mathrm{red}}\right)}^{\prime }\sigma_{s}^{2}+Z_{d}^{\delta }G{\left(Z_{d}^{\mathrm{red}}\right)}^{\prime }\sigma_{s}^{2} \end{array}$$

are all zero matrices. Thus, $Q_{\mathrm{all}}=Q_{\mathrm{red}}+\mathrm{Var}\left(m_{s}\right)+\mathrm{Var}\left(m_{d}\right)$, and thus, the variance Var(y) is identical in the classical gametic model and the reduced model with gametic relationships. Both models also have identical expectations of y, so they are equivalent, q.e.d.

### Appendix A4: Maternal genetic variance in a reduced model

We consider a reduced equation for a single observation:

$$y_{i}=\mu +m_{d}+a_{d}^{d}+a_{s}^{s}+r_{i}.$$

This equation comprises the maternal breeding value $m_{d}$ of the dam d of individual i, as well as the transmitting ability as dam $a_{d}^{d}$ of the dam d of i, the transmitting ability as sire $a_{s}^{s}$ of the sire s of i, and the residual $r_{i}$.

Then, the covariance of the respective vectors of random genetic effects is:

$$\mathrm{Var}\left[\begin{array}{c} m\\ a^{s}\\ a^{d} \end{array}\right]=\left[\begin{array}{ccc} \sigma_{m}^{2} & \sigma_{ms} & \sigma_{md}\\ \sigma_{ms} & \sigma_{s}^{2} & \sigma_{sd}\\ \sigma_{md} & \sigma_{sd} & \sigma_{d}^{2} \end{array}\right]⊗\frac{1}{2}A,$$

where $\sigma_{m}^{2}$ is the maternal gametic variance. We use $\frac{1}{2}A$ as the relationship matrix (assuming that all records are from final progeny), so the incidence matrix for maternal breeding values needs to have nonzero entries of two to match this set of covariances.

The variance of observations has the nonresidual component:

$$Q_{rm}=\left[\begin{array}{ccc} Z_{m} & Z_{s} & Z_{d} \end{array}\right]\left[\begin{array}{ccc} \sigma_{m}^{2} & \sigma_{ms} & \sigma_{md}\\ \sigma_{ms} & \sigma_{s}^{2} & \sigma_{sd}\\ \sigma_{md} & \sigma_{sd} & \sigma_{d}^{2} \end{array}\right]⊗\frac{1}{2}A\left[\begin{array}{c} Z_{m}{}^{\prime }\\ Z_{s}{}^{\prime }\\ Z_{d}{}^{\prime } \end{array}\right],$$

which includes the incidence matrices $Z_{m}$, $Z_{s}$, and $Z_{d}$ that link observations to maternal genetic effects, transmitting abilities as sire, and transmitting abilities as dam, respectively. $Q_{rm}$ is a sum of nine matrices and the following matrix equalities can be found by dropping the respective components of variance and replacing $Z_{m}$ by ${\tilde{Z}}_{m}=\frac{1}{2}Z_{m}$:

$$\begin{array}{c} {\tilde{Z}}_{m}A{\tilde{Z^{\prime }}}_{m}=Z_{d}A{Z^{\prime }}_{d}={\tilde{Z}}_{m}A{Z^{\prime }}_{d}=Z_{d}A{\tilde{Z^{\prime }}}_{m}\\ {\tilde{Z}}_{m}A{Z^{\prime }}_{s}=Z_{d}A{Z^{\prime }}_{s}\\ Z_{s}A{\tilde{Z^{\prime }}}_{m}=Z_{s}A{Z^{\prime }}_{d} \end{array}.$$

An underlying fact is that the incidence matrices $Z_{m}$ and $Z_{d}$ link all observations to genetic effects of the same animals, *i.e.*, of the dam of each final progeny. Thus, the incidence matrices $\frac{1}{2}Z_{m}=Z_{d}$ are equal, and they constitute the equalities according to the equations above. Consequently, $Q_{rm}$ can be rewritten as:

$$Q_{rm}=\frac{1}{2}\left[\begin{array}{c} Z_{m}A{Z^{\prime }}_{m}\sigma_{m}^{2}+Z_{d}A{Z^{\prime }}_{d}\sigma_{d}^{2}+Z_{m}A{Z^{\prime }}_{d}\sigma_{md}+Z_{d}A{Z^{\prime }}_{m}\sigma_{md}\\ Z_{m}A{Z^{\prime }}_{s}\sigma_{ms}+Z_{d}A{Z^{\prime }}_{s}\sigma_{sd}\\ Z_{s}A{Z^{\prime }}_{m}\sigma_{ms}+Z_{s}A{Z^{\prime }}_{d}\sigma_{sd}\\ Z_{s}A{Z^{\prime }}_{s}\sigma_{s}^{2} \end{array}\right],$$

which is as follows in terms of the incidence matrices of the reduced model without maternal genetic effects:

$$Q_{rm}=\frac{1}{2}\left[\begin{array}{cc} Z_{d}A{Z^{\prime }}_{d}\left(\sigma_{d}^{2}+4\sigma_{m}^{2}+4\sigma_{md}\right) & Z_{d}A{Z^{\prime }}_{s}\left(\sigma_{sd}+2\sigma_{ms}\right)\\ Z_{s}A{Z^{\prime }}_{d}\left(\sigma_{sd}+2\sigma_{ms}\right) & Z_{s}A{Z^{\prime }}_{s}\sigma_{s}^{2} \end{array}\right].$$

Therefore, the variance in the transmitting ability as dam and the covariance with the transmitting ability as sire are contaminated by components of the maternal genetic (co-)variances. Thus, in the presence of maternal genetic effects, $\sigma_{d}^{2}$ and $\sigma_{s}^{2}$ cannot be inferred from the reduced model. Moreover, we cannot correctly calculate the weights of the observations because this would require knowledge of these two components of variance.

Interestingly, we can assume the absence of genomic imprinting and utilize $\sigma_{g}^{2}=\sigma_{s}^{2}=\sigma_{d}^{2}=\sigma_{sd}$, and thus the residual variance of observation i becomes:

$$\frac{1}{2}\left(1-F_{s,i}\right)\sigma_{g}^{2}+\frac{1}{2}\left(1-F_{d,i}\right)\sigma_{g}^{2}+\sigma_{e}^{2}.$$

Consequently, the imprinting variance becomes:

$$\sigma_{i}^{2}=\left(\sigma_{d}^{2}+4\sigma_{m}^{2}+42\sigma_{md}+\sigma_{s}^{2}\right)-2\left(\sigma_{sd}+2\sigma_{ms}\right)=4\sigma_{m}^{2}.$$

### Appendix A5: Maternal variance in a classical gametic model

The model equation for a single observation $y_{i}$ in a gametic model with maternal effects is:

$$y_{i}=\mu +g_{d,1}^{m}+g_{d,2}^{m}+g_{i,1}^{s}+g_{i,2}^{d}+e_{i},$$

which includes the maternal effect (superscript m) of the paternal (1) gamete $g_{d,1}^{m}$ and the maternal (2) gamete $g_{d,2}^{m}$ of the dam d of individual i, while $g_{i,1}^{s}$ is the effect of the paternal gamete of individual i as sire, $g_{i,2}^{d}$ is the effect of the maternal allele of individual i as dam, and $e_{i}$ is the residual. The covariance of random gametic effects is:

$$\mathrm{Var}\left[\begin{array}{c} g^{m}\\ g^{s}\\ g^{d} \end{array}\right]=\left[\begin{array}{ccc} \sigma_{m}^{2} & \sigma_{ms} & \sigma_{md}\\ \sigma_{ms} & \sigma_{s}^{2} & \sigma_{sd}\\ \sigma_{md} & \sigma_{sd} & \sigma_{d}^{2} \end{array}\right]⊗G.$$

The covariance of the vector of observations y is $V=Q_{mm}+I\sigma_{e}^{2}$, with

$$Q_{mm}=\left[\begin{array}{ccc} Z_{m} & Z_{s} & Z_{d} \end{array}\right]\left[\begin{array}{ccc} \sigma_{m}^{2} & \sigma_{ms} & \sigma_{md}\\ \sigma_{ms} & \sigma_{s}^{2} & \sigma_{sd}\\ \sigma_{md} & \sigma_{sd} & \sigma_{d}^{2} \end{array}\right]⊗G\left[\begin{array}{c} Z_{m}{}^{\prime }\\ Z_{s}{}^{\prime }\\ Z_{d}{}^{\prime } \end{array}\right].$$

All other components of the covariance are as defined above in Appendix A4.

$Q_{mm}$ is a sum of six matrices where three are related to variances: $Z_{m}G{Z^{\prime }}_{m}\sigma_{m}^{2}$, $Z_{s}G{Z^{\prime }}_{s}\sigma_{s}^{2}$, and $Z_{d}G{Z^{\prime }}_{d}\sigma_{d}^{2}$. The other three contributions depend on covariances: $Z_{m}G{Z^{\prime }}_{s}\sigma_{ms}+Z_{s}G{Z^{\prime }}_{m}\sigma_{ms}$, $Z_{m}G{Z^{\prime }}_{d}\sigma_{md}+Z_{d}G{Z^{\prime }}_{m}\sigma_{md}$, and $Z_{s}G{Z^{\prime }}_{d}\sigma_{sd}+Z_{d}G{Z^{\prime }}_{s}\sigma_{sd}$. In contrast to the reduced model, the incidence matrices $Z_{m}$ and $Z_{d}$ relate the records to different gametic effects, and thus they are not equal. Therefore, all six addends of $Q_{mm}$ are linearly independent and all components of the variance can be separated.
